# Inside the April 2024 Issue

**DOI:** 10.24908/pocus.v9i1.17638

**Published:** 2024-04-22

**Authors:** Benjamin T Galen

**Affiliations:** 1 Department of Medicine, Albert Einstein College of Medicine and Montefiore Medical Center Bronx, NY USA

**Keywords:** POCUS Journal, Commentary

Dear Readers,

We are thrilled to bring you the first issue of the ninth volume of POCUS Journal. Published since 2016, POCUS Journal is the only multi-disciplinary, peer-reviewed, POCUS-focused journal that is free for authors and readers alike. We are grateful for the vision of our founder Dr. Amer Johri and for the support of CINQUILL Medical Publishers, Inc. as we enter our ninth year of publication. POCUS is an ever-changing field as clinicians seek out better and faster solutions for patient care at the bedside. We at POCUS Journal continue to evolve as well. Our editorial board is growing to meet the demands of our high volume and high-quality manuscript submissions. We welcome Dr. Andre Kumar of Stanford University and Dr. Manpreet Malik of Emory University to the Internal Medicine section. We also welcome Dr. Andrea Matho of the University of Southern California to the Pediatrics section. Natalie Kearn joins us from Queen’s University as the Social media Editor. If you haven’t seen her high-yield infographics on social media I highly encourage you to visit POCUS Journal on X and Instagram for summaries of research articles and other content published in our journal. A journal our size also needs help with statistics, and we are excited that Nicholas Grubic has joined our team from the University of Toronto as Statistical Editor. We also have a new Editorial Director of Artificial Intelligence, Dr. Bredon Crawford. We are the first journal to feature an AI bot on our website, which is thanks to Dr. Crawford. Its name is “PJ” and you can find it on the bottom right of our website: www.pocusjournal.com. And finally, we are excited to have had Kathryn Matsushita join our team as Copyeditor, helping us preserve our standard of high quality publishing as the volume of submitted articles and size of our issues continues to grow. 

**Figure 1:**
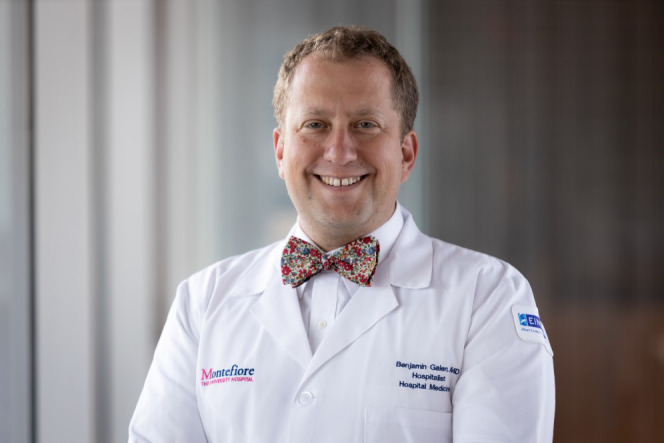
Dr. Benjamin T. Galen, Editor-in-Chief, POCUS Journal

With the addition of these new members, I also share the bittersweet news that our Managing Editor Braeden Hill will be leaving POCUS Journal to pursue MD/PhD studies at The University of Toronto. Braeden started at POCUS Journal in 2020 as Social Media Editor and his contributions these past few years have been remarkable. We welcome Laura Guzman of Queen’s University into the role of Managing Editor.

Every issue I oversee at POCUS Journal strikes me as better than the last, with fascinating cases and important research that answers key questions related to point of care ultrasound. This issue is no exception. 

Please find our author guidelines here: https://pocusjournal.com/author-guidelines/

Sincerely,

Benjamin T. Galen, MD

Department of Medicine, Albert Einstein College of Medicine and Montefiore Medical Center, Bronx, NY

Editor-In-Chief , POCUS Journal

